# The development of cross-cultural recognition of vocal emotion during childhood and adolescence

**DOI:** 10.1038/s41598-018-26889-1

**Published:** 2018-06-14

**Authors:** Georgia Chronaki, Michael Wigelsworth, Marc D. Pell, Sonja A. Kotz

**Affiliations:** 1 Developmental Cognitive Neuroscience (DCN) Laboratory, School of Psychology, University of Central Lancashire, Preston, UK; 2 Division of Neuroscience & Experimental Psychology, University of Manchester, Manchester, UK; 3 School of Psychology, University of Southampton, Southampton, UK; 4 Manchester Institute of Education, University of Manchester, Manchester, UK; 5 School of Communication Sciences and Disorders, McGill University, Montréal, QC Canada; 6 Department of Neuropsychology, Max-Planck Institute for Human Cognitive and Brain Sciences Leipzig, Leipzig, Germany; 7 Faculty of Psychology and Neuroscience, Department of Neuropsychology and Psychopharmacology, Maastricht University, Maastricht, Netherlands

## Abstract

Humans have an innate set of emotions recognised universally. However, emotion recognition also depends on socio-cultural rules. Although adults recognise vocal emotions universally, they identify emotions more accurately in their native language. We examined developmental trajectories of universal vocal emotion recognition in children. Eighty native English speakers completed a vocal emotion recognition task in their native language (English) and foreign languages (Spanish, Chinese, and Arabic) expressing anger, happiness, sadness, fear, and neutrality. Emotion recognition was compared across 8-to-10, 11-to-13-year-olds, and adults. Measures of behavioural and emotional problems were also taken. Results showed that although emotion recognition was above chance for all languages, native English speaking children were more accurate in recognising vocal emotions in their native language. There was a larger improvement in recognising vocal emotion from the native language during adolescence. Vocal anger recognition did not improve with age for the non-native languages. This is the first study to demonstrate universality of vocal emotion recognition in children whilst supporting an “in-group advantage” for more accurate recognition in the native language. Findings highlight the role of experience in emotion recognition, have implications for child development in modern multicultural societies and address important theoretical questions about the nature of emotions.

## Introduction

Vocal cues provide a rich source of information about a speaker’s emotional state. The term ‘*prosody’* derives from the Greek word ‘*prosodia’* and refers to the changes in pitch, loudness, rhythm, and voice quality corresponding to a person’s emotional state^1,2^. Recent debates have focused on whether the ability to recognise vocal emotion is universal (e.g., due to biological significance to conspecifics) or whether it is influenced by learning, experience, or maturation^3,4^.

It is argued that humans have an innate, core set of emotions which seem to be expressed and recognised universally^5^. However, the way emotional expressions are perceived can be highly dependent on learning and culture^6^. It has been argued that when attending to the prosody conveyed in speech, listeners apply universal principles enabling them to recognise emotions in speech from foreign languages as accurately as their native language^7^. However, it is also argued that cultural and social influences create subtle stylistic differences in emotional prosody perception^3^. In addition, cultural influences may impact on how listeners interpret emotional meaning from prosody^8^. This is known as an “in-group advantage” enabling listeners to recognise emotional expressions in their native language more accurately than in a foreign languages^7^.

Previous research has provided support for the hypothesis of an “in-group advantage” in the recognition of vocal emotional expressions. Recent studies by Pell and colleagues^9^ used pseudo-utterances produced by Spanish, English, German, and Arabic actors in five different emotions (anger, disgust, fear, sadness and happiness) as well as neutral expressions. Pseudo- utterances reduce the effect of meaningful lexical-semantic information on the perception of vocally expressed emotions and mimic the phonotactic and morpho-syntactic properties of the respective language. The emotion can therefore only be recognised by the prosody in the speech. Monolingual native listeners of Spanish listened to the series of pseudo-utterances and were asked to judge the emotional state of the speaker in each language. Pell and colleagues^9^ found that participants could accurately recognise all emotions from foreign languages at above chance level. In addition, participants were significantly better at recognising emotions when listening to utterances spoken in their native language, Spanish, compared to other foreign languages^9^. Scherer and colleagues^10^ have found the same pattern of results when presenting pseudo-utterances spoken by four German actors conveying emotions of fear, sadness, anger, and joy to native German speakers and native speakers of eight other languages. In addition, Scherer and colleagues argued that linguistic similarity between cultures influenced vocal emotion recognition^10^. Thompson and Balkwill^11^ provided supporting evidence for the “in-group advantage” by showing that English participants, who identified emotions conveyed by speakers of English, Chinese, Japanese, German, and Tagalog, could recognise emotions in their native language better than in foreign languages^11^. However, this study did not find that linguistic similarity influenced vocal emotion recognition, contradicting findings by Scherer and colleagues^10^. More recently, a study comparing English and Hindi listeners, extended these findings by showing that emotion recognition in a non-native language is less accurate as well as less *efficient*; they reported an “in-group advantage” in both accuracy and speed of vocal emotion recognition in each of their cultural groups, despite the fact that Hindi participants were second language speakers of English^12^ (see also^13^). Collectively, these data argue that the ability to recognise vocally expressed emotions is a universal ability but it is also dependent on cultural and linguistic influences, supporting the theory that both nature and nurture may contribute to vocal emotion recognition.

It is noteworthy that all existing studies in this area have focused on adults and no studies to date have been conducted in children. This is surprising given the prominent role of vocal emotions in children’s social interactions. Sensitivity to vocal emotion has been associated with individual differences in social competence^14^ and behaviour problems^15^ in children.

Emotions have been argued to be relational and functional (e.g., serving a purpose) and are embedded in social communicative relationships throughout development^16^. New-borns respond to the valence of speech prosody produced in their mother’s native language but not in nonmaternal languages^17^. Studies examining three-month-olds in interaction with their mothers have shown that the dyads who were positively aroused emotionally vocalized in synchrony. These behaviours have been argued to contribute to the formation of mother-infant bonds^18^. More recent research has shown that vocal mimicry and synchrony facilitate emotional and social relationships in 18-month-old infants^19^. At 12-months, infants can distinguish among different negative emotions but may not be differentially responsive to discrete negative emotional signals^20,21^. Self-conscious emotions (embarrassment, shame, guilt) begin to develop between 15 and 18 months^22^. Children develop awareness of multiple emotions as early as 5 to 6 years of age^23^. During the preschool years children begin to have an understanding that others have intentions, beliefs and inner states^24–26^. From 7 to 10 years children develop appreciation of norms of expressive behaviour and use of expressive behaviour to regulate relationship dynamics and close friendships. Awareness of one’s own emotions (i.e., guilt about feeling angry) begins to develop during the adolescent years^16^. Research has shown that emotion awareness is an important factor for adaptive empathic reactions in 11–16 year olds^27^. Similar work has shown that facial emotion recognition reached adult levels by 11 years whereas vocal emotion recognition continued to develop at 11 years^28^.

Facial emotion processing develops with age and its developmental course depends on the type of emotion^28,29^. Happiness and sadness have been shown to be accurately recognised from facial expressions by children as young as 5 and 6 years of age with accuracy levels close to adult levels. By the time children reached 10 years of age, they have acquired the ability to recognise fear, anger and neutrality from faces and their ability to recognise disgust reached adult levels at 11 or 12 year of age^29^. Other studies have shown that sadness recognition from facial expressions was delayed across development compared to anger and happiness in 4-11-year-old children^28^. Awareness of multiple emotions and cognitive construction of one’s emotional experience takes place later in development. Young adolescents from 10 years of age can integrate contrasting emotions about the same person^30^. An increase in competence to recognise facial expressions of disgust and anger has been found from mid to late puberty^31^. Neuroimaging studies suggest an increase of amygdala response to emotional facial expressions during adolescence relative to other ages^32^. Adolescents tend to be more attuned to peer’s facial emotions, as indicated by studies showing higher accuracy to recognise expressions of peer-aged stimuli compared to adult stimuli in 13-year-olds^33^. Under conditions of greater task difficulty, however, adolescents showed performance deficits comparable to those of adults. This may suggest that more fine-grained aspects of facial emotion recognition continue to develop beyond adolescence^34^. Research has shown a female advantage at facial emotion recognition in infants, children and adolescents^35^. These findings can be interpreted in relation to models on both neurobiological maturation and socialization as important factors in the development of sex differences in emotion recognition skills.

Vocal emotion processing has early developmental origins. Infants can discriminate among vocal expressions soon after birth^36^ and tend to display more eye opening responses to happy voices than to angry, sad, or neutral voices^17^. Previous research has found that 4- and 5-year-old children were less accurate to recognize sentences with angry, happy, and sad tone of voice compared to 9- and 10-year-olds^37,38^. Recognition of surprise but no other emotions improved with age in 5-10-year olds in a study asking children to match simple (e.g., anger) and complex (e.g., surprise) emotions from non-verbal vocalisations to photographs of people^39^. Recognition of emotion from speech continues to develop and reaches adult-like levels at about 10 years of age^40^. More recent research examining the development of emotion recognition from non-linguistic vocalisations has shown that vocal emotion recognition improves with age and continues to develop in early adolescence^28^. Sadness perception from non-linguistic vocalisations followed a slower developmental trajectory compared to recognition of anger and happiness from the preschool years until 11 years of age^28^. In the same study, 6–9-year-olds did not differ significantly from 10-11-year-olds in recognising angry, happy, and sad vocal expressions. A similar study found no improvement with age in the perception of emotional speech (angry, happy, sad, and neutral) across a number of tasks in 9- to 15-year-olds^41^. Research using a vocal emotion recognition task in 6–11-year-old children has identified differential event-related brain potentials to distinct vocal expressions of emotion (angry, happy, and neutral)^42^. Paralinguistic emotion recognition can be considered part of language acquisition. Research has shown a slight female advantage in 4–6-year-olds in several linguistic domains^43^. Similarly, girls were slightly ahead of boys in early communicative gestures, in productive vocabulary and in combining words^44^. In the study by Lange and colleagues^43^ sex differences in language skills seemed to vanish around 6 years. The same study found that boys varied more than girls in their language competence. Other studies have found equal variance in language skills for girls and boys below the age of 3^45,46^.

Despite recent advances in the development of vocal emotion recognition during childhood, research of cross-cultural vocal emotion recognition in children remains extremely limited. This is surprising considering the increasing diversity and multiculturalism of contemporary societies^47^. Research has shown that children are exposed to many foreign languages in their daily social interactions in Western societies^48^. In addition, research into the development of cross-cultural emotion recognition from vocal expressions can address fundamental theoretical questions about the nature of emotions and the extent to which emotion perception is determined by universal biological factors or socio-cultural factors or their interaction. Some researchers have argued that experience-independent maturational processes may be implicated in the development of emotion recognition^49^. Others have suggested that early experience may interact with neurobiological structures to determine the development of emotion recognition^50^.

Existing studies in children’s cross-cultural emotion recognition have relied exclusively on facial stimuli. A study presented Chinese and Australian children aged 4, 6, and 8 years with Chinese and Caucasian (American) facial expressions of basic emotions and asked children to choose the face that best matched a situation. Results showed that 4-year-old Chinese children were better than Australian children at choosing the facial expression that best fit the situation in Chinese faces^51^. In a similar study, Gosselin and Larocque^52^ presented Caucasian and Asian (Japanese) faces of basic emotions to 5–10-year-old French Canadian children and read to the children short stories describing one of the basic emotions. Children were asked to choose the face that best fit the emotion in the story. Results showed that children displayed equal levels of accuracy for Asian and Caucasian faces but performance was influenced by the emotion type. Specifically, children recognised fear and surprise better from Asian faces, whereas disgust was better recognised from Caucasian faces^52^. Findings suggest some influence of facial characteristics from different ethnicities on emotion recognition. Overall, findings from existing studies using facial stimuli suggest that cross-cultural differences in emotion recognition may be present in early childhood. Learning to recognise emotions develops as children acquire greater experience with language. More accurate recognition of emotion in native language with development suggests greater influence of culture-specific factors and experience on emotion recognition.

Recent research has highlighted links between emotional information processing and behaviour problems in children. Individual differences in hyperactivity and conduct problems have been negatively associated with recognition of angry, happy, and sad vocal expressions since the preschool years^15^. This is consistent with studies using facial emotion stimuli^53^. School-aged children with Attention-Deficit/Hyperactivity Disorder have shown atypical neural response, in terms of enhanced N100 amplitude, to vocal anger^54^. Emotional problems have also been associated with poor emotion recognition. Individuals with high trait anxiety are more likely to interpret others’ emotions from face-voice pairs in a negative manner^55^. Similarly, participants who were induced with a feeling of stress before a vocal emotion recognition task performed worse than non-stressed participants^56^. Emotion recognition and emotion regulation jointly predicted intercultural adjustment in university students; specifically, recognition of anger and emotion regulation predicted positive adjustment while recognition of contempt, fear and sadness predicted negative adjustment^57^. Despite evidence that behavioural and emotional problems negatively affect interpersonal sensitivity to emotion, previous research has not examined links between individual differences in behavioural and emotional problems and cross-cultural vocal emotion recognition in children. Since children with behavioural and emotional problems show lower sensitivity to social cues of emotion within their own cultures, it is possible that cultural influences on emotion recognition would be relatively small in this group of children.

The first aim of the current study was to investigate whether there is an “in-group advantage” in vocal emotion recognition in childhood. Studying cross-cultural vocal emotion recognition during development can contribute to a better understanding of the extent to which these abilities are shaped by learning and experience or are universal and biologically determined abilities. Building on adult work, we hypothesised that English children would recognise vocal emotions from foreign language with above chance performance but would also show an “in-group advantage” enabling more accurate recognition of emotion from the native language. The second aim of this study was to examine the developmental trajectory of cross-cultural differences in vocal emotion recognition. We aimed to answer the question of whether vocal emotion recognition improves throughout development as children acquire greater exposure to their native language. We predicted that improvement in vocal emotion recognition with development would be larger in the native language.

Finally, based on research showing associations between vocal emotion recognition and individual differences in personality and behaviour traits in adults^58^ and children^15^, we explored the impact of behavioural and emotional problems as well as emotion regulation on vocal emotion recognition. We predicted that vocal emotion recognition would be positively associated with emotion regulation and negatively associated with behavioural and emotional problems.

## Data Processing

Raw data were transformed into measures of accuracy according to the two high threshold model^59^. This model has been used in previous studies examining vocal emotion recognition accuracy in children^15,28^.

*Discrimination accuracy* (Pr) is defined as sensitivity to discriminate an emotional expression and is given by the following equation: Pr = ((number of hits + 0.5)/(number of targets + 1)) − ((number of false alarms + 0.5)/(number of distractors + 1))^59^. Pr scores take values which tend to 1, 0 and −1 for accuracy at better than chance, close to chance and worse than chance respectively. For example, in our task with 10 trials of each of the 5 conditions (angry, happy, sad, fearful, neutral) × 4 languages per emotion (English, Spanish, Arabic, Chinese), amounting to 200 trials in total, if a child classified 8 angry voice as angry but he/she also classified as angry, 4 happy voices, 4 sad voices, 2 fearful voices and 3 neutral voices and 0 for all other expressions, then his/her accuracy for angry voices would be: ((8 + 0.5)/(10 + 1)) − ((4 + 4 + 2 + 3 + 0 + 0.5)/(40 + 1)) = 0.44, suggesting that his/her accuracy for angry voices is better than chance. Our measure of discrimination accuracy took into account not only the stimuli identified correctly (hits) but also all possible misidentifications (e.g., non-angry expressions classified as angry). This is similar with the Hu scores^60^ used in the studies by Pell and colleagues^7,9^ to correct for differences in item frequency among categories and individual participant response biases. As in our study, the Hu scores also take into account not only the stimuli identified correctly (hits) but also possible misidentifications (e.g. non-angry expressions classified as angry).

## Results

Kolmogorov-Smirnov tests confirmed that data met assumptions for parametric analysis. Discrimination accuracy for voices was significantly different from chance for children [*t* (25) > 0.11, *p* < 0.001], adolescents [*t* (32) > 0.10, *p* < 0.001], and adults [*t* (21) > 0.12, *p* < 0.001] across all emotions. Results did not change when repeating the analyses for each emotion × language condition. Independent-samples t-tests showed statistically significant differences between boys and girls in discrimination accuracy. Cohen’s d estimates of effect sizes are reported for the t-test comparisons. Specifically, males presented significantly lower scores than females for accuracy to recognize sad English voices [t (77) = −2.68, p = 0.009, *d* = 0.60], happy Spanish voices [t (77) = −2.34, p = 0.020, *d* = 0.04], and sad Chinese voices [t (77) = −2.44, p = 0.017, *d* = 0.55] in the whole sample, and angry English voices [t (24) = −2.87, p = 0.009, *d* = 1.12] in the child sample.

Scores of discrimination accuracy were entered into a mixed-design ANOVA with Emotion (angry, happy, sad and fear) and Language (English, Spanish, Chinese, Arabic) as within-subject factors and Age group (children, adolescents, adults) as the between-subject factor. Main effects and interaction terms were broken down using simple contrasts. Significant effects emerging from the one-way ANOVAs, whenever relevant, were followed up through Tukey’s (HSD) post-hoc comparisons (p < 0.01). For post-hoc comparisons, we also report Cohen’s d estimates of effect sizes which can take values ranging from small (*d* = 0.2) to medium (*d* = 0.5), and large (*d* = 0.8)^61^. Because neutral stimuli are not emotional and served as filler items in the experiment, and for consistency with previous work in adults^7^, neutral scores were not entered in the main analysis to focus on effects of basic emotions^62^. Nevertheless, to examine effects of language on the recognition of neutral stimuli, a one-way ANOVA was performed on the accuracy scores for neutral voices. This analysis showed a significant effect of language (*F* (3, 228) = 119.07, *p* < 0.001, ${\eta }_{p}^{2}$ = 0.61). Post hoc tests indicated that neutral expressions were recognized significantly better in English and Chinese than Arabic (*F* (1, 76) = 227.40, *p* < 0.001, ${\eta }_{p}^{2}$ = 0.75, see also Tables 1–4). Cohen’s d effect size for the difference between English and Arabic was 1.73 and between Chinese and Arabic was 1.83.

**Table 1:** Mean (SD) of discrimination accuracy for vocal expressions per age group, language and emotion.

	English				Chinese				Spanish				Arabic			
	Angry	Happy	Sad	Fear	Angry	Happy	Sad	Fear	Angry	Happy	Sad	Fear	Angry	Happy	Sad	Fear
Children	0.74(0.17)	0.40(0.24)	0.60(0.15)	0.50(0.23)	0.60(0.18)	0.22(0.18)	0.54(0.20)	0.35(0.19)	0.55(0.19)	0.27(0.16)	0.11(0.18)	0.19(0.19)	0.15(0.13)	0.17(0.11)	0.33(0.19)	0.25(0.19)
Adolescents	0.74(0.16)	0.45(0.25)	0.57(0.20)	0.50(0.22)	0.58(0.13)	0.24(0.18)	0.48(0.26)	0.42(0.21)	0.46(0.22)	0.32(0.17)	0.14(0.15)	0.18(0.15)	0.16(0.13)	0.14(0.12)	0.34(0.18)	0.14(0.13)
Adults	0.87(0.10)	0.80(0.12)	0.88(0.06)	0.80(0.11)	0.66(0.10)	0.30(0.16)	0.70(0.12)	0.63(0.14)	0.53(0.16)	0.60(0.19)	0.22(0.15)	0.34(0.18)	0.16(0.12)	0.33(0.19)	0.60(0.15)	0.57(0.16)

Note 1: Accuracy: −1 = worse than chance, 0 = chance, 1 = better than chance, Note 2: Children (8–10 years), Adolescents (11–13 years), Adults (19–35 years).

**Table 2:** Mean percentage (SD) of vocal expressions classified correctly (in bold) and misclassifications in children.

Language	Emotion	Children’s’ response				
		Angry	Happy	Sad	Fear	Neutral
English	Angry	**89**.**6**(**12**.**7**)	2.8(6.1)	2.0(4.0)	2.4(5.2)	3.2(6.9)
	Happy	13.6(17.7)	**46**.**4**(**26**.**0**)	21.6(19.5)	8.8(9.3)	9.6(10.9)
	Sad	4.0(8.1)	3.2(6.9)	**81**.**2**(**18**.**3**)	9.6(13.4)	2.0(5.0)
	Fear	12.4(14.8)	2.8(6.8)	22.0(17.5)	**58**.**4**(**23**.**2**)	4.4(11.2)
	Neutral	14.0(15.3)	10.80(12.2)	23.6(16.8)	3.20(5.57)	**48**.**4**(**27**.**8**)
Chinese	Angry	**68**.**4**(**20**.**3**)	9.6(16.7)	5.6(8.2)	7.2(9.8)	9.2(9.0)
	Happy	15.6(12.9)	**29**.**5**(**20**.**8**)	11.6(12.8)	17.2(15.4)	26.4(20.5)
	Sad	2.0(4.0)	2.4(5.2)	**72**.**8**(**19**.**4**)	15.6(15.5)	7.2(8.9)
	Fear	2.8(5.4)	6.0(8.6)	28.0(13.5)	**47**.**6**(**21**.**0**)	15.6(15.3)
	Neutral	3.2(6.30)	10.4(12.4)	14.4(16.3)	5.6(9.6)	**66**.**4**(**21**.**9**)
Spanish	Angry	**68**.**8**(**22**.**0**)	8.4(12.1)	4.0(6.4)	5.6(9.1)	13.2(12.1)
	Happy	16.0(12.20)	**33**.**20**(**15**.**7**)	9.20(10.98)	10.4(10.9)	30.8(19.0)
	Sad	11.2(14.8)	5.2(9.2)	**30**.**0**(**20**.**6**)	15.6(15.0)	38.0(23.6)
	Fear	4.8(6.5)	4.0(7.6)	32.8(11.0)	**27**.**6**(**21**.**6**)	30.8(19.8)
	Neutral	10.4(15.1)	6.8(9.4)	31.2(20.3)	6.0(6.4)	**45**.**6**(**22**.**4**)
Arabic	Angry	**19**.**0**(**14**.**0**)	2.4(5.2)	24.0(16.3)	13.2(11.4)	44.4(26.1)
	Happy	2.0(5.0)	**20**.**0**(**14**.**0**)	26.0(12.2)	12.0(11.1)	40.0(17.3)
	Sad	3.6(9.0)	1.6(0.4.7)	**66**.**0**(**21**.**9**)	13.2(14.0)	15.6(13.2)
	Fear	4.0(5.8)	7.2(10.2)	32.8(13.4)	**32**.**8**(**13**.**4**)	17.2(16.7)
	Neutral	1.6(3.7)	6.0(8.6)	39.2(15.5)	14.4(17.3)	**38**.**8**(**17**.**1**)

**Table 3:** Mean percentage (SD) of vocal expressions classified correctly (in bold) and misclassifications in adolescents.

Language	Emotion	Adolescents’ response				
		Angry	Happy	Sad	Fear	Neutral
English	Angry	**85**.**0**(**15**.**2**)	5.9(11.6)	1.87(3.9)	3.7(5.5)	3.4(6.0)
	Happy	6.5(8.6)	**55**.**9**(**27**.**4**)	15.0(17.4)	8.4(13.2)	14.0(13.6)
	Sad	3.4(7.8)	1.8(4.7)	**73**.**4**(**22**.**3**)	13.4(13.2)	7.8(10.4)
	Fear	8.7(10.0)	4.3(8.4)	19.6(10.3)	**61**.**8**(**21**.**8**)	5.3(9.5)
	Neutral	5.0(7.6)	14.3(16.0)	17.5(12.4)	6.5(7.8)	**56**.**5**(**25**.**8**)
Chinese	Angry	**69**.**6**(**14**.**9**)	6.5(10.0)	5.3(9.15)	8.7(9.0)	9.6(10.0)
	Happy	25.3(20.3)	**30**.**9**(**20**.**5**)	11.2(12.1)	12.1(10.4)	20.3(15.3)
	Sad	2.8(6.3)	2.8(5.8)	**64**.**0**(**25**.**8**)	20.0(15.4)	10.3(11.7)
	Fear	2.5(5.6)	5.3(7.1)	23.7(17.2)	**55**.**3**(**22**.**4**)	13.1(11.5)
	Neutral	3.7(8.3)	16.2(13.3)	12.1(12.8)	5.6(7.1)	**62**.**1**(**25**.**6**)
Spanish	Angry	**55**.**3**(**23**.**5**)	11.2(12.1)	9.3(12.9)	7.5(10.5)	16.5(11.5)
	Happy	10.3(10.6)	**40**.**0**(**17**.**2**)	9.6(10.6)	9.3(12.9)	30.6(14.1)
	Sad	7.8(10.0)	4.3(8.0)	**34**.**6**(**18**.**3**)	12.1(13.3)	40.9(19.0)
	Fear	5.0(6.0)	8.1(13.3)	33.7(18.6)	**25**.**6**(**13**.**9**)	27.5(20.1)
	Neutral	6.8(10.3)	6.2(8.7)	33.1(20.2)	6.5(9.3)	**47**.**1**(**21**.**1**)
Arabic	Angry	**17**.**5**(**14**.**6**)	6.5(7.4)	24.6(12.4)	15.6(13.1)	35.6(18.3)
	Happy	4.0(6.6)	**19**.**7**(**14**.**0**)	19.3(12.9)	11.9(10.6)	45.0(14.1)
	Sad	2.5(5.0)	4.0(9.1)	**61**.**5**(**24**.**0**)	13.4(14.5)	17.8(17.3)
	Fear	3.7(9.0)	8.7(10.0)	22.1(14.9)	**46**.**5**(**21**.**2**)	18.7(.14.5)
	Neutral	3.4(7.0)	8.4(12.4)	35.3(18.4)	9.6(13.3)	**43**.**1**(**19**.**7**)

**Table 4:** Mean percentage (SD) of vocal expressions classified correctly (in bold) and misclassifications in adults.

Language	Emotion	Adult’s’ response				
		Angry	Happy	Sad	Fear	Neutral
English	Angry	**91**.**0**(**13**.**7**)	4.5(9.7)	0(0)	0(0)	4.5(7.5)
	Happy	2.3(5.6)	**86**.**4**(**14**.**0**)	1.1(3.2)	0.6(2.4)	9.4(12.4)
	Sad	0(0)	0(0)	**95**.**0**(**5**.**6**)	1.7(3.2)	1.7(3.2)
	Fear	4.1(8.7)	1.1(3.3)	8.2(10.0)	**84**.**7**(**14**.**0**)	1.7(3.9)
	Neutral	2.9(5.8)	1.7(3.9)	7.0(9.2)	0.6(2.4)	**87**.**6**(**12**.**5**)
Chinese	Angry	**77**.**6**(**16**.**0**)	8.2(8.8)	2.3(4.3)	8.8(12.1)	2.9(4.7)
	Happy	27.6(21.6)	**36**.**4**(**23**.**1**)	1.7(3.9)	11.7(8.8)	22.3(17.5)
	Sad	0(0)	0.6(2.4)	**78**.**2**(**15**.**5**)	10.6(8.2)	10.5(13.9)
	Fear	1.1(3.3)	2.9(5.8)	12.9(13.1)	**75**.**3**(**18**.**7**)	7.6(13.9)
	Neutral	1.7(5.2)	10.0(12.2)	2.9(5.8)	1.1(3.3)	**84**.**1**(**17**.**7**)
Spanish	Angry	**60**.**0**(**22**.**6**)	20.0(14.1)	0.5(2.4)	1.7(3.9)	17.6(16.8)
	Happy	5.3(7.1)	**68**.**2**(**25**.**5**)	2.9(5.8)	2.9(4.7)	20.6(23.8)
	Sad	8.2(8.8)	0(0)	**36**.**5**(**23**.**4**)	10.5(9.9)	46.4(20.9)
	Fear	0.6(2.4)	0.6(2.4)	35.8(20.0)	**41**.**7**(**20**.**6**)	16.4(19.9)
	Neutral	3.5(7.8)	0.6(2.4)	39.4(14.3)	0.6(2.4)	**55**.**8**(**14**.**16**)
Arabic	Angry	**15**.**3**(**16**.**2**)	4.1(5.0)	15.8(11.7)	5.3(6.2)	60.4(21.3)
	Happy	0(0)	**35**.**8**(**25**.**2**)	11.7(10.7)	3.5(6.0)	48.8(23.9)
	Sad	0(0)	0.6(2.4)	**84**.**7**(**17**.**7**)	1.1(3.3)	13.5(16.9)
	Fear	1.7(3.9)	2.9(5.8)	22.9(14.5)	**62**.**9**(**20**.**8**)	9.4(8.9)
	Neutral	0(0)	1.7(3.9)	28.8(20.8)	1.7(7.2)	**67**.**6**(**20**.**7**)

**Figure 1:**
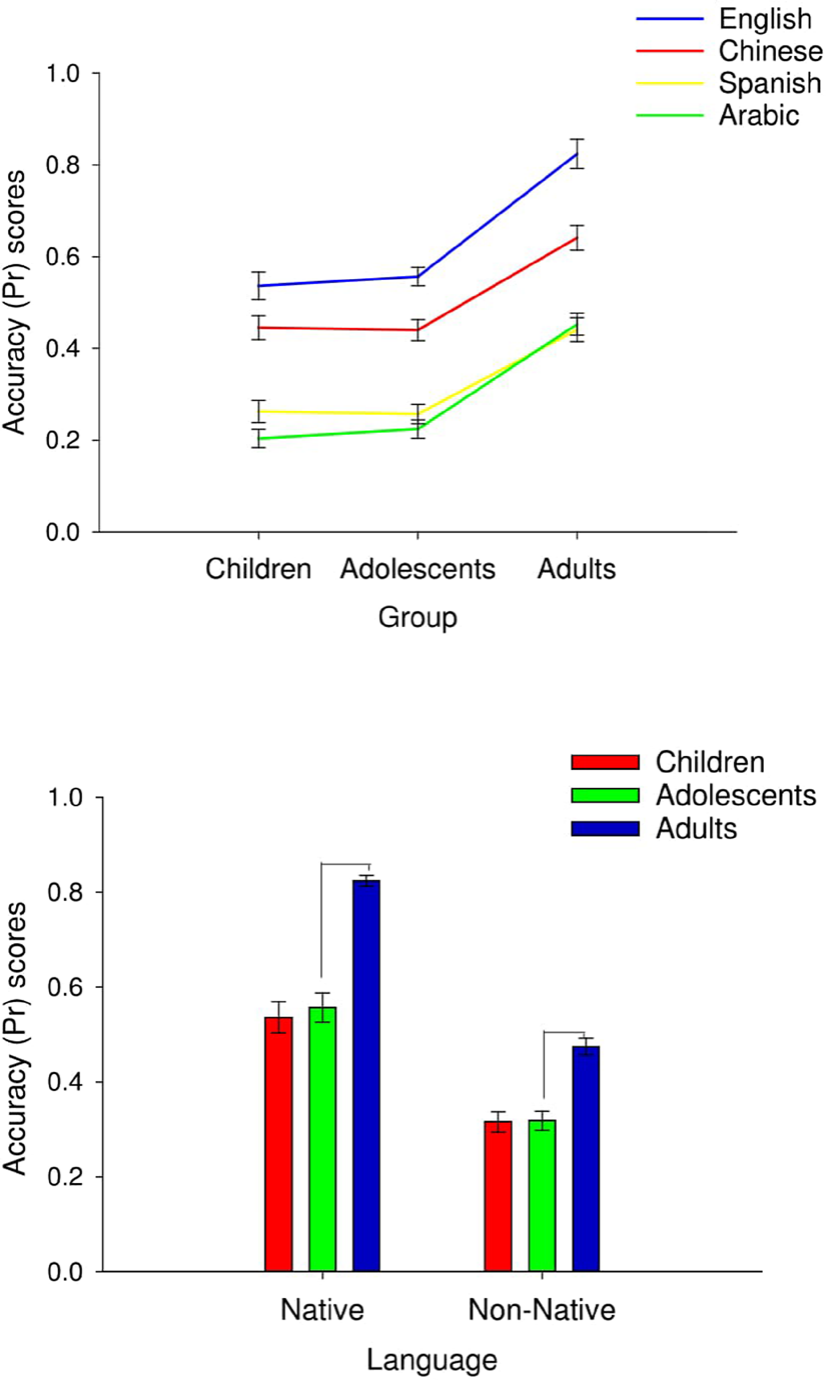
Top panel: Line graph with error bars showing the mean accuracy (Pr) scores for each language per age group. Bottom panel: Bar graph with error bars showing larger improvement in vocal emotion recognition accuracy between adolescents and adults for the native language (0 = chance, 1 = perfect performance).

Table 1 displays means and standard deviations for accuracy for vocal expressions by emotion, language, and age. Misattribution patterns between emotions are presented in Tables 2–4. There was a significant main effect of language on accuracy (*F* (3, 228) = 321.08, *p* < 0.001, ${\eta }_{p}^{2}$ = 0.80). Contrasts showed that English participants performed significantly better when recognising vocal emotions in their native language (English) than in each of the three foreign languages (p < 0.001, Cohen’s *d* = 0.74, 1.85 and 1.98 for English compared to Chinese, Spanish and Arabic respectively). Participants were also more accurate to recognise Chinese compared to Spanish (*d* = 1.19) and Arabic (*d* = 1.35), and less accurate to recognise Arabic compared to Spanish (p < 0.001, *d* = 0.20), as shown in Fig. 1.

**Figure 2:**
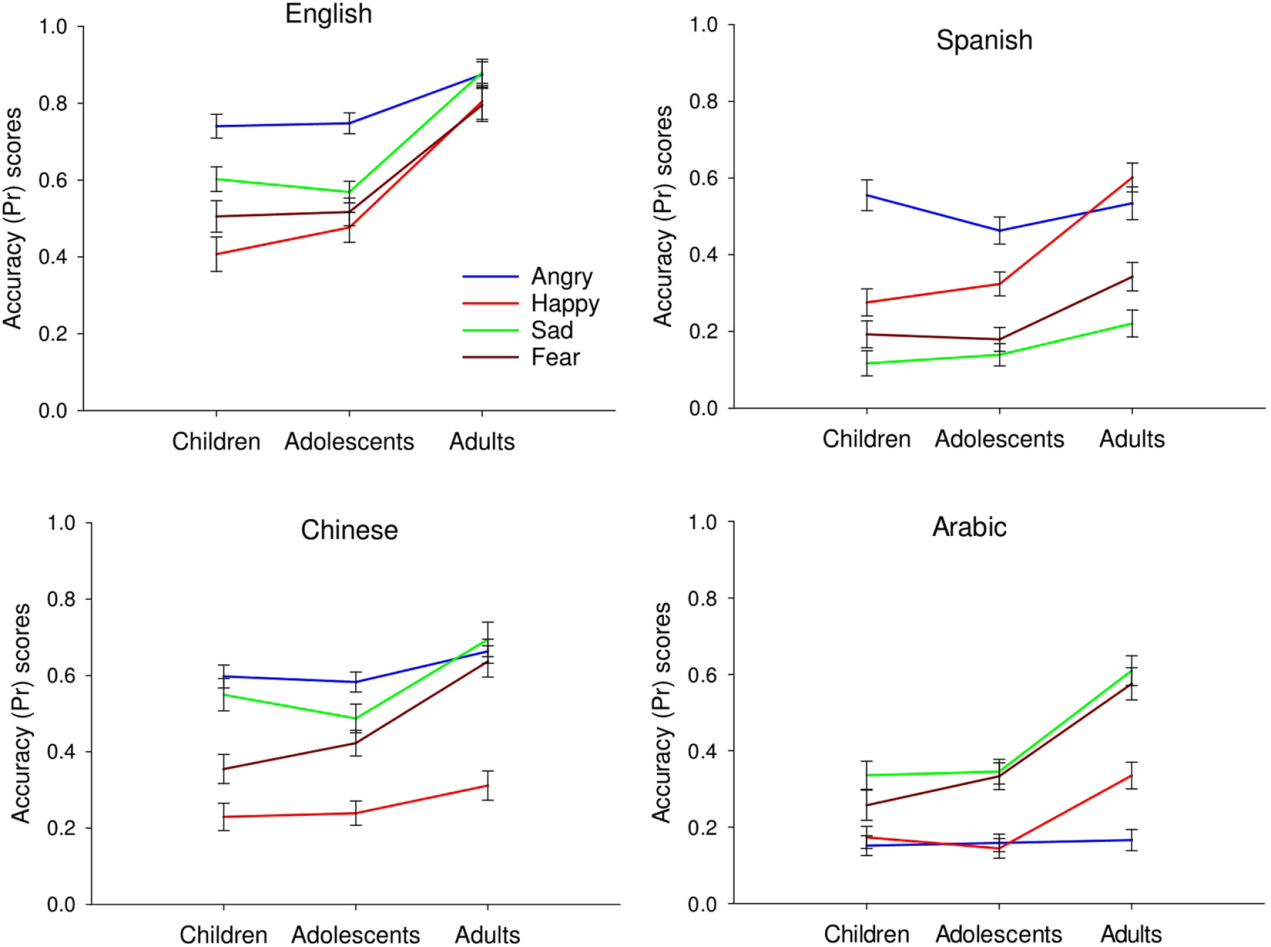
Line graph with error bars showing the mean accuracy (Pr) scores for each language, emotion and age group (0 = chance, 1 = perfect performance).

There was a significant main effect of age on accuracy (*F* (2, 75) = 23.78, *p* < 0.001, ${\eta }_{p}^{2}$ = 0.38). Adults were significantly more accurate to recognise vocal expressions of emotion compared to children and adolescents (p < 0.001, *d* = 2.31 and 2.18 respectively) who did not differ from each other. Emotion had a significant main effect on accuracy (*F* (2, 228) = 43.16, *p* < 0.001, ${\eta }_{p}^{2}$ = 0.36). Participants were more accurate for angry and sad voices compared to fear (*d* = 0.62) and more accurate for fear and sad compared to happy (*d* = 0.45 and *d* = 0.22 respectively). They were also less accurate for happy and sad compared to anger (all ps < 0.001, *d* = 1.27 and *d* = 0.40 respectively). The language effect varied by emotion type (*F* (9, 684)^language × emotion^ = 88.70, *p* < 0.001, ${\eta }_{p}^{2}$ = 0.54), as shown in Fig. 2. These results are presented in the supplementary material because they are of less theoretical interest here (see Supplement 2).

The age effect varied by emotion type (*F* (3, 228)^emotion × age^ = 7.60, *p* < 0.001, ${\eta }_{p}^{2}$ = 0.16), as shown in Fig. 2. For angry expressions, there was no significant difference in accuracy between the age groups (p > 0.05). However, adults were significantly more accurate than children and adolescents for happy (p < 0.001, *d* = 1.90), sad (p < 0.001, *d* = 1.70) and fear (p < 0.001, *d* = 1.57), with no significant difference in accuracy between children and adolescents (p > 0.05).

The age effect also varied by language type (*F* (6, 228)^language × age^ = 4.20, *p* < 0.001, ${\eta }_{p}^{2}$ = 0.10). Adults were significantly more accurate to recognise vocal expressions of emotion than children and adolescents (who did not differ from each other) and this difference was more pronounced for English which followed a steeper developmental trajectory compared to the other languages (p < 0.001, *d* = 2.36).

Results also showed a significant language × emotion × age interaction effect on accuracy (*F* (9, 684)^language × emotion × age^ = 88.70, *p* < 0.001, ${\eta }_{p}^{2}$ = 0.54), as shown in Fig. 2. To explore this we ran additional analyses in which accuracy scores of the language x emotion conditions were entered in One-Way ANOVA examining the effect of emotion and language on accuracy for the age groups separately. Post-hoc Tukey’s comparisons indicated that for English, children and adolescents were significantly less accurate compared to adults for all emotion types and especially happiness (p < 001, *d* = 1.83), sadness (p < 0.001, *d* = 2.27) and fear (p < 0.001, *d* = 1.72). For the non-native languages (Spanish, Chinese and Arabic), however, children and adolescents were not significantly different from adults for angry expressions (p > 0.05). In addition, no significant difference was found between the two child groups and adults for sad Spanish (p > 0.05) and happy Chinese (p > 0.05) expressions.

To simplify the results and because our aim was to examine developmental effects on recognition accuracy for native compared to non-native language, we conducted a further ANOVA with accuracy for native and non-native language per emotion as the dependent measure and age as a between subjects factor. We did this by combining scores of all non-native languages per emotion and comparing them with recognition scores of the native language. Overall, the largest improvement was observed between adolescence and adulthood, as shown in Fig. 1. Improvement in vocal emotion recognition from adolescence to adulthood was larger for the native language (p < 0.001, *d* = 2.36) relative to the non-native language (p < 0.001, *d* = 1.76), as shown in Fig. 1. Results showed that developmental trajectories of emotion recognition differed as a function of language type. For the native language, recognition accuracy improved with age for all emotions (*F* (2, 78) > 5.68, *p* < 0.005); children and adolescents were less accurate than adults. For the non-native language, however, there was no improvement in the recognition of anger with age (*F* (2, 78) = 1.37, *p* = 2.60). As above, we used Cohen’s d estimates of effect sizes ranging from small (*d* = 0.2) to medium (*d* = 0.5), and large (*d* = 0.8)^61^. Cohen’s d effect size for the difference between native and non-native language across emotions in the overall sample was 1.73 which is indicative of a large effect size.

### Vocal emotion recognition and behaviour

Pearson’s correlations examined associations between vocal emotion recognition for native and non-native language across emotions and interpersonal variables (behavioural and emotional problems, emotion regulation and cognitive reappraisal) in the whole sample of children and adults separately. These analyses controlled for age because age was significantly associated with recognition accuracy for native and non-native language (r = 0.56, p < 0.001). Because we were not interested in emotion-specific patterns but rather the overall relationship between recognition from native and non-native language and behaviour, we collapsed across emotions for these analyses. We report emotion-specific patters in Supplement 3. Results showed that conduct problems in children were negatively associated with recognition accuracy from their native language (r = −0.27, p = 0.040). In addition, emotional problems in children were negatively associated with recognition accuracy from non-native language (r = −27, p = 0.045). In adults cognitive reappraisal was negatively associated with recognition accuracy for the non-native language (r = −0.43, p = 0.045). No other associations were significant (p > 0.05).

## Discussion

### Language and emotion effects on vocal emotion recognition

This is the first study to examine the development of vocal emotional recognition in foreign languages in children and adolescents. Children recognised vocal emotions at above chance levels in all three tested foreign languages. In addition, English children were more accurate when recognising vocal emotions in their native language. Children were more accurate for angry and sad voices compared to happiness and fear. Emotion-related effects on accuracy were different for the different languages tested. Accuracy improved with age especially for happiness, sadness, and fear. Age-related improvement was more prominent for the native language. Accuracy improved with age for all emotions in the native language, but not in the non-native language where improvement was not observed for certain emotions (e.g., anger).

First, the overall recognition rates per language in our study are consistent with previous studies in adults. The mean recognition rates for the stimuli, which were selected for the current study based on previous studies, was as follows: English (97.46%), Chinese (93%), Spanish (81.12%) and Arabic (71.90%, see^7,9^). Similarly, in our study, the highest mean recognition was for English (93.0%) followed by Chinese (74%), Spanish (62.50%), and Arabic (61.10%) in adults and English (68.90%), Chinese (54.50%), Spanish (40.00%), and Arabic (35.20%) in children and adolescents. Consistent with previous studies, our study showed that English was recognised with the highest accuracy rate and Arabic with the lowest rate. This mirrors results from the validation study by Pell and colleagues^7^, in which a total of 91% items were retained for English but only 49% of items were retained for Arabic. Arabic was also perceived by most participants (92%) as the most difficult language condition for recognising emotions in a post-session questionnaire after the recognition task^7^. In the study by Pell and colleagues^9^ with Spanish speakers overall emotion recognition scores ranged from 64% in Spanish, 58% in English, and 50% in Arabic. In the study by Liu & Pell^63^ with Chinese speakers, only items which reached a recognition consensus rate of three times chance performance (42%) per emotion were included in the validation database. This is consistent with previous literature, which has shown that vocal emotions are recognized at rates approximately four times chance^1,64^. In summary, accuracy rates in our study are stable and consistent with previous research, suggesting the existence of similar inference rules from vocal expressions across languages.

Second, emotion effects on accuracy in our study are similar to those reported in adults^9^. We found higher accuracy for angry and sad voices compared to happiness and fear and higher accuracy for fear and sad compared to happiness. This is consistent with Pell and colleagues^7^ who found that anger, sadness and fear tended to result in higher recognition rates across languages compared to expressions of happiness. Liu and Pell^63^ also found that fear had the highest recognition, followed by anger, sadness, and happiness. Our study and previous work converge towards a general advantage for recognising negative emotions. This is compatible with evolutionary theories arguing that vocal cues are associated with threat and need to be highly salient to ensure human survival^65–67^. In both our study and previous studies^7,10,64^ accuracy was especially low when participants were asked to recognise happy expressions. Our results are consistent with previous research showing that although happiness is recognisable more easily from facial expression^68^, it is more difficult to recognise in vocal expressions^3,69^. In contrast, negative emotions (i.e., anger) are often poorly recognised from the face but best recognised from the voice.

Third, the error confusion patterns between emotions in our study are consistent with those of previous adult studies. In our study, participants showed a tendency to confuse sadness and fear and a tendency to categorize neutral expressions as sad. Happiness was also mislabelled as neutral in many cases. This is consistent with results from previous studies in adults. Studies by Pell and colleagues^7,9^ showed that participants tended to confuse sadness or anger with neutral expressions and to categorize fear as sadness, although these patterns were not uniform across languages. The most frequent systematic error observed in adult studies was that neutral expressions were mislabelled as conveying sadness. In addition, fear was confused with sadness in English, Hindi and Arabic. Happiness was misjudged as neutral in English and Arabic^7^. Similarly, in the study by Scherer and colleagues^10^, fear was frequently confused with sadness and sadness with neutral. These recognition rates, including the low recognition accuracy for happiness, are similar to previous research using the same stimulus material^64^ and to recognition rates obtained with a larger set of different actors and emotion portrayals^1^.

In summary, a systematic analysis of recognition rates per language and emotion as well as confusion matrices from our study shows striking similarities with data from previous adult studies, suggesting that recognition rates in our study are stable and likely to generalise to new samples.

Our study revealed significant emotion × language interactions. Specifically, vocal expressions of anger compared to fear were significantly more accurately recognised in English than in Arabic. Expressions of fear, which were recognized relatively poorly when compared to other emotions in other languages, were significantly more accurately recognised in Arabic. In addition, fear compared to happiness was significantly more accurately recognised in Chinese than English. Vocal expressions of happiness compared to fear were significantly more accurately recognised in Spanish than in Chinese. A clear processing advantage for happiness when produced in Spanish is consistent with findings from previous studies in Spanish speaking individuals^9^. In addition, Pell and colleagues^9^ found that sadness was recognised with the least accuracy in Spanish which was significantly lower than Arabic and English. Fear showed no significant difference in recognition accuracy across languages^7^. Scherer and colleagues^10^ showed that correlations between accuracy rates for different emotions among languages indicated uniformly high correlations, suggesting that recognition of different emotions is highly comparable across cultures. In the same study, the error patterns were similar across cultures (German, French, English, Italian, Spanish and Indonesian), suggesting similar inference rules from vocal expressions across cultures. In summary, both our study and previous work^9–11^ seem to converge to cross-language tendencies to recognise vocal emotion, with happiness being the emotion showing a clear processing advantage from Spanish across studies. However, future studies should use encoders from a number of languages, rather than only one language and construct an encoder-decoder emotion matrix to systematically examine the intercultural encoding and decoding of vocal emotion.

Our finding that children recognised vocal emotions at above chance levels in all tested foreign languages extends previous work in adults^7^. These findings support the claim that vocal emotions contain pan-cultural perceptual properties which allow accurate recognition of basic emotions in a foreign language. To our knowledge, our study is the first to show that the ability to recognise emotions from the tone of voice is a universal ability which is already in place in middle childhood. Importantly, emotion recognition was specific to vocal rather than linguistic aspects given that we used pseudo-sentences which did not contain meaningful linguistic content. The finding that cross-cultural vocal emotion recognition is an early developing mechanism is compatible with theories on the universality of emotional expressions within humans and continuity of emotion across species^70–72^. It also supports nativist-oriented theories of development arguing in favor of the innate nature of differentiated emotional expressions^73,74^. Supporting evidence derives from studies showing that deaf and blind children display expressions of anger and happiness in suitable situations even though they could not have learned these emotions through experience^75^. Recent fMRI research has found that the human brain shows remarkable functional specialisation for processing emotional information from human voices already at 3 months of age^76^. In summary, the above view partly challenges the role of experience and learning in vocal emotion recognition.

Although children recognised vocal emotions at above chance levels in all foreign languages, they were more accurate to recognise emotions in their native language (English). The effect size for the comparison between native and non-native language (*d* = 1.70 and *d* = 5.24 for children and adolescents respectively) was found to exceed Cohen’s convention^61^ for a large effect (*d* = 0.80). This finding suggests that vocal emotion recognition is influenced to some extent by cultural and social factors. Children and adolescents recognised emotions from their native language at rates similar to those reported in adult studies, especially for anger and sadness (80–90%)^7^. These findings support the hypothesis of an “in-group advantage”^7^, highlighting the role of socio-cultural norms (e.g., ‘display rules’) learnt by social interactions in emotion recognition^77^. From a developmental perspective, this finding is consistent with models highlighting the motivational and communicative nature of emotional expressions^78–81^. These models have assumed that the development of emotion recognition is predominantly experience-reliant. Consistent with this idea, research has shown that children’s acquisition of emotion-descriptive language is anchored in relationship contexts^82^. Parent-child relationships have been found to play an important role in children’s acquisition of emotion understanding^83^. Our study extends previous work by showing that although there is a universal ability to recognise vocal emotions, the way emotions are recognised is also influenced by cultural aspects. Therefore, social and biological determinants may interact to form an understanding of emotions throughout development, and theories considering one determinant (biological versus social) in isolation cannot account for the whole picture in the development of vocal emotion recognition. Our findings are compatible with an integrated model with biological maturation playing an important early role and socialization maintaining biologically based predispositions with regard to vocal emotion recognition.

It is important to note that consistent with previous research we found a slight female advantage in vocal emotion recognition^35^. In the adult literature, female judges have been found to present slightly better vocal emotion recognition rates than male judges^10^. A female advantage in emotion recognition should be considered in the context of sex-different evolutionary selection pressures related to survival and reproduction^84^. For example, a female advantage in the appraisal of vocal emotion can be attributed to evolutionary pressure to detect subtle changes in infant signals^85^. A female advantage can be explained by sex-different maturational rates. Females seem to mature faster than males and early maturation is associated with better verbal abilities^86^. Language-related sex differences may be affected by biological factors and hormonal effects^87^. It has also been argued that the development of sex differences in emotion recognition may depend on the interaction of maturational and experiential factors^35^. Girls may present biological predispositions to an emotion recognition advantage which is amplified in situations of eliciting experiences. Research has shown that emotion scripts and acquisition of emotion concepts can merge with gender socialization. For example, Fivush found that mothers of 3-year-olds tended to talk in a more elaborated fashion about sadness with their daughters and more about anger with their sons^88^. Similarly, by using more varied emotional language in conversations with daughters, parents socialised girls to be more attuned to the emotions of others^89^.

Although accuracy was higher for the native language (English) than a foreign language, accuracy for recognising emotions in Chinese was also higher compared to Spanish or Arabic. Based on previous research showing that linguistic similarity has a positive impact on the ability to recognise emotions in a foreign language^10^ we would expect that English native speakers would be more accurate when recognising emotions expressed in another European language such as Spanish rather than Chinese. Our findings seem to be more consistent with findings by other researchers^7,11^ showing that linguistic similarity does not influence vocal emotion recognition.

The studies by Pell and colleagues^7^ have provided limited evidence that acoustic or perceptual patterns vary systematically as a function of similarity among different language structures (‘linguistic similarity’). Pell and colleagues^7^ have systematically analysed the acoustic parameters of pseudo-sentences from different languages and have found that speakers of English, German and Arabic exploit acoustic parameters of fundamental frequency, duration and intensity in relatively equal measure to differentiate a common set of basic emotions. Signalling functions may be dictated by modal tendencies independent of language structure^7^. Results from the studies by Pell and colleagues are consistent with previous work^11^ which did not find that linguistic similarity influenced vocal emotion recognition. In contrast, Scherer and colleagues^10^ asked judges from nine countries in Europe, the United States, and Asia to recognise language-free vocal emotion portrayals by German actors and found that accuracy decreased with increasing language dissimilarity from German. Specifically, the rank order of countries with respect to overall recognition accuracy mirrored the decreasing similarity of languages. The lowest recognition rate was reported for the only country studied that did not belong to the Indo-European language family: Indonesia^10^. Given that non-linguistic stimuli were employed, it is possible that effects may be due either to segmental information (e. g, phoneme-specific fundamental frequency, articulation differences, formant structure) or to suprasegmental parameters (e.g. prosodic cues of intonation, rhythm and timing). Consequently, future research should examine potential influences of linguistic similarity on vocal emotion recognition in children.

### An evolutionary approach to vocal emotions

In interpreting our findings, one should consider evolutionary perspectives to emotion. In our study, recognition accuracy was higher for angry and sad voices compared to fear and higher for fear and sad compared to happy. It has been argued that emotions evolved because they promoted specific actions in life-threatening situations and therefore increased the odds of survival^65^. For example, the self-protection system focuses attention on specific sensory cues (e.g., angry faces) which elicit the emotional response of fear which facilitates behavioural escape from a perceived danger^90^. This response activates knowledge structures and cognitive associations into working memory^91^. It has been argued that human threat management systems are biased in a risk-averse manner, erring toward precautionary responses even when cues only inquisitively imply threat^65^. Activation of the self-protection system may cause perceivers to mistakenly perceive anger in faces^92^. For instance, even when someone about to attack often looks angry, sometimes he may be simply posing. This signal detection problem has been argued to produce errors which tend to be predictably biased in a direction that is associated with reduced costs to reproductive fitness^93^. Whereas models based on specific action tendencies provide compelling accounts of the function of negative emotions (anger, sadness), positive emotions do not normally arise in life-threatening situations and do not seem to create urges to pursue a specific course of action^94^. Positive emotions (happiness) have been argued to serve less prominent evolutionary functions relative to negative emotions, such as anger and sadness^94^.

In addition, our study showed that although accuracy improved for all emotions for the native language, no improvement in the recognition of anger with age was evident for the non-native language. Based on the above framework, this may suggest that the functional structure of the emotion of ‘anger’ evolved to match the evolutionary summed structure of its target situations which were culture-specific. It has been argued that certain selection pressures caused genes underlying the design of an adaptation to increase in frequency until they became species-typical or stably persistent in a particular environment^66^. The conditions that characterise an environment of evolutionary adaptedness are argued to represent a constellation of specific environmental regularities that had a systematic impact which endured long enough for evolutionary change^66^. It is possible that improvement in the recognition of anger cannot be cultivated in a non-native and non-culture specific environment.

Evolutionary approaches to emotions have suggested that emotions (including anger) are designed to solve adaptive problems that arose during human evolutionary history^95^. According to these models, emotions relate to motivational regulatory processes the human brain is designed to generate and access. Cognitive programs that govern behaviour evolve in the direction of choices that lead to the best expected fitness payoffs. Emotion programs guide the individual into appropriate interactive strategies. For example, fear will make it more difficult to attack a rival whereas anger will make it easier. Individuals make efforts to reconstruct models of the world so that future action can lead to payoffs. For example, happiness is an emotion that evolved to respond to the condition of unexpectedly good outcomes. Similarly, anger is the expression of a functionally structured system whose design features and subcomponents evolved to regulate thoughts, motivation, and behaviour in the context of resolving conflicts of interest in favour of the angry individual^96,97^. It is likely that anger is an adaptation designed by natural selection given its universality across individuals and cultures^98,99^. The study of cross-cultural similarities in emotion recognition can help us generate a holistic picture of human life history, in other words, a ‘human nature’.

### Age effects on vocal emotion recognition

This research demonstrates for the first time a developmental pattern of cross-cultural vocal emotion recognition. Specifically, we showed striking improvement in vocal emotion recognition from adolescence to adulthood with smaller improvement in accuracy between childhood and adolescence. This highlights the importance of adolescence as an important milestone for the development of vocal emotion recognition skills consistent with our previous work^28^. Although previous research has suggested high adaptability of the nervous system in early development (e.g. infancy, preschool years) in relation to emotion^100^ and language^101^ skills, our findings show larger improvement during adolescence than childhood. This may suggest that plasticity for emotion processing skills is higher at later developmental stages. However, our study has not tested adolescents older than 13 years and this leaves open the possibility that late adolescence may be associated with greater improvement compared to early adolescence^102^. Similar work has shown that emotional prosody is difficult to interpret for young children and that prosody does not play a primary role in inferring others’ emotions before adolescence^103^. In particular, prosody did not enable children to infer emotions from others at age 5, and this skill was still not fully mastered at age 13^103^. Similarly, our study has not tested children younger than 8 years to examine the early developmental origins of these skills.

Our study showed a steeper developmental profile in recognising vocal emotion from the native language (English) compared to a foreign language. It is possible that vocal emotion recognition improves throughout development as individuals acquire greater exposure to their native language. In addition, our study demonstrated emotion-specific developmental trajectories in the recognition of vocal emotion from foreign language. Vocal emotion recognition continued to improve from adolescence to adulthood for all emotion types when emotions were expressed in the native language. For Spanish, Chinese, and Arabic, however, no improvement was found with age for angry voices and similarly for sad Spanish and happy Chinese voices. Although, accuracy for sad Spanish and happy Chinese voices was generally low across age groups, which might explain the lack of age-dependent changes, results demonstrate a more extended developmental trajectory for recognition of vocal emotions from the native language compared to a foreign language. The finding that recognition continues to improve from adolescence to adulthood across all emotion types for the native language only, may suggest that vocal emotion recognition is dependent more heavily on socio-cultural factors during the period from adolescence to adulthood. Future research should examine the neural mechanisms underlying vocal emotion recognition during this critical period in development.

According to life history theory, individuals face a number of evolutionary challenges related to survival and reproduction and emotions enable individuals to cope with these challenges. In adolescence elaborated vocal behaviours played a role in courtship and intersexual competition^104^. An important function emerging in adolescence is social talking (speech in which the topic is other people), which is prominent in females, and a tendency to tease peers, which is prominent in males^105^. These functions facilitate achievement of goals that are important for adolescents, such as status and relationships. Social relationships influence personal and social identify in adolescents. An effective way to signal affiliation in adolescence and increasing autonomy is through linguistic markers, particularly phonetic and vocal cues. Adolescents not only manipulate language but also revise it. At a phonological level, changes of complex articulation serve to identify members of social groups^106^. At sexual maturity, vocal and verbal performances increased fitness by facilitating attainment of social rank and mating relationships^107^. It has been argued that important aspects of language not only do not develop until adolescence, but cannot do so because the biological functions associated with that stage played an evolutionary role in their construction^104^. Adolescence is characterised by marked improvements in pragmatics -inference of speakers’ emotions and intentions^108^. Verbally performative behaviours (e.g., ‘verbally showing off’) tend to blossom during adolescence. Youths begin to acquire in-group slang expressions, use metaphors, jokes and sarcasm and engage in rapid humorous verbal exchanges. Performance deficits related to vocal behaviour in adolescence have been linked with negative social consequences and feelings of loneliness^109–111^.

Improvements in the ability to recognise emotion from voices during adolescence may be related to increasing exploratory behaviour and exposure to novel vocal cues during this period in life. It is also important to take into account that the ‘social brain’, defined as the network of brain regions responsible for understanding others’ mental states, undergoes substantial functional and structural development during adolescence^112,113^. Face-processing abilities and the brain systems that support them continue to show age-related changes between adolescence and adulthood^113^. There is striking lack of evidence in the development of neural networks underlying vocal emotion recognition in adolescence and how the environment influences this development. Educational policies tend to emphasize the importance of early childhood social skills interventions. However, training vocal emotion recognition skills at later developmental stages, such as adolescence, may yield greater improvement if we consider that these skills develop more rapidly during this period, as supported by our findings.

### Vocal emotion recognition and behaviour

Consistent with our predictions, the current study demonstrated a negative relationship between vocal emotional recognition and behavioural and emotional problems. Childhood externalising behaviour (conduct problems) was associated with lower accuracy to recognise negative emotions, especially anger, from the native language. This is consistent with our previous work in children^15^. Childhood internalising behaviour (emotional problems) was negatively associated with recognition accuracy from the non-native language. We did not find a strong pattern of associations between behaviour variables and vocal emotion recognition from the native language compared to a foreign language, suggesting that the relationship between behaviour and vocal emotion recognition is not dependent on socio-cultural factors. Findings are in line with previous research in adults^58^ and extend current research by demonstrating that culture-specific factors may not influence the relationship between vocal emotion recognition and behaviour and emotional problems. However, the low levels of symptoms in children from the general population in our study, when combined with high levels in performance, may not have allowed clear associations between childhood behaviour problems and vocal emotion processing difficulties to emerge.

It is important to consider potential factors for individual differences and socialization of emotion. Research has shown that parents who were better coaches of their children’s emotions had children who understood emotions better^114^. References to feeling states made by mothers when their child was 18 months, were associated with the child’s speech about feeling states at 24 months^115^. Similar research showed that family discourse about feelings at 36 months was associated with children’s ability to recognise emotions at 6 years, independently of children’s verbal ability^116^. Research has linked social class with the context in which feeling states are discussed in families^117^. Middle-class mothers discussed more complex concepts than did working-class mothers during a block building construction task^118^. In addition, middle class parents have been found to be more affiliative in their conversational styles than working class parents although no differences in children’s speech were found as a result of social class^119^.

To ensure effects were not due to task difficulty (influencing accuracy), all children were asked to give verbal confirmation they understood the task. In addition, all children successfully completed a number of practice trials before taking part in the task. Further, we carefully selected well validated stimuli^7^ to ensure that age effects cannot be attributed to stimuli properties. Specifically, we selected those stimuli with the highest accuracy rates from previous adult studies, and accuracy rates in this study were similar to those in previous adult work (see Supplement 1).

A limitation of the current study is the relatively small sample size. Further work with larger samples is necessary. In our study, a minimum of 22 participants were recruited per age group; while this sample is typical of comparable studies in the literature^7,11,63^, a larger sample size could further improve the reliability of our data. Importantly though, our results were stable and consistent with previous adult research, suggesting they are likely to generalizable to new samples. In addition, emotional expressions were based on portrayals from professional actors and actors with experience with public speaking. However, professional actors may vary in their abilities to encode vocal emotions^120^. Despite our efforts to focus our analyses on stimuli which were representative of a specific emotion category, it is possible that individual abilities in encoding the vocal emotions may have contributed to our results. A related limitation at the stage of encoding the vocal stimuli used in our study was that while actors of English tended to have acting experience, most Arabic encoders tended to have experience in public speaking^7^. This may have contributed to the tendency for lower recognition rates for Arabic compared to English. A similar analysis by Scherer and colleagues^10^ has shown that within each emotion there was variation of recognition accuracy for specific stimuli, suggesting that some stimuli were less typical or extreme. However, it should be noted that the inclusion of less typical stimuli has been argued to increase the sensitivity for the detection of intercultural differences in emotion recognition^10^. Future studies should also employ longitudinal designs to understand age-related changes in vocal emotion recognition. An important target for future research would be to track the development of vocal emotion recognition beyond 13 years of age. Future work should also consider recruiting younger children to establish how early the ability to recognise emotions from foreign language develops. In the present study we did not test children younger than 8 years because previous research has not found significant differences in vocal emotion recognition between 6 and 8 years^28^. Similarly, we did not test children younger than 6 years because pre-schoolers have been shown to perform poorly in vocal emotion recognition tasks^28^. Finally, future studies should extend current findings to children who are native speakers of Chinese, Spanish, and Arabic.

Despite the above limitations the present study showed that both maturation and socialization factors (and their interaction) are important in the development of vocal emotion recognition. Adolescence may provide a possible ‘window of opportunity’ for learning vocal emotional skills. This may be facilitated in appropriate socio-cultural environments. Building on knowledge that vocal emotion recognition skills develop over the course of adolescence and are susceptible to social factors, future intervention efforts might be more effective when targeting vocal emotion recognition skills during this period and take into account social influences.

## Methods

### Participants

Eighty monolingual individuals (57 children and 22 young adults) participated in the study, as shown in Tables 5 and 6. All participants were native English speakers and had no previous experience with speakers of Spanish, Chinese, and Arabic as established by self-reports and school records. Participants were not included in the study if they had a diagnosis of attention-deficit/hyperactivity disorder, autism, dyslexia, or other disorder based on self-reports and school records. Children were recruited from primary and secondary schools and were selected from two age groups based on previous developmental research in vocal emotion recognition^28^. Adult participants consisted of University students. Child assent and adult informed consent were obtained prior to participation. The study was approved by the University of Manchester Ethics Committee. All methods were performed in accordance with the relevant guidelines and regulations at the University of Manchester, UK.

**Table 5:** Participants per age group.

Age group	Age range	Mean	SD	N
Children	8.5–10.5	9.50	0.80	25 (15 males)
Adolescents	11.0–13.0	12.30	0.65	32 (19 males)
Adults	19.0–35.0	23.00	5.40	22 (8 males)

**Table 6:** Participants’ behavioural characteristics and verbal knowledge scores.

	Children (n = 25)		Adolescents (n = 32)		Adults (n = 22)	
	M	SD	M	SD	M	SD
Verbal knowledge	35.80	4.60	38.60	6.20	37.20	5.10
Behaviour						
Hyperactivity	2.00	1.60	2.00	1.30	6.20	2.45
Inattention	1.60	1.30	2.24	1.50	6.80	3.05
Conduct problems	2.10	1.90	2.00	1.95	—	—
Emotional problems	2.70	1.80	3.45	2.40	3.00	3.45
Emotion Regulation						
Reappraisal	21.00	4.30	20.90	3.60	28.30	7.45
Suppression	11.50	3.10	11.40	2.40	12.50	5.20

Note: Self-reports based on SDQ for children and adolescents, CBS and GHQ for adults and ERQ for children and adults. Verbal knowledge as measured by WISC and WAIS for children and adults respectively. The range of scores is as follows: Child hyperactivity, inattention, conduct problems and emotional problems: 0–10, Adult Hyperactivity and inattention: 0–27, Adult emotional problems: 0–12, Child reappraisal: 6–30, Child suppression: 4–20, Adult reappraisal: 6–42, Adult suppression: 4–36. WISC: 0–68.WAIS:0–57.

### Materials

#### Vocal stimuli validation

The stimuli consisted of emotional ‘pseudo-utterances’ produced by native speakers of four different languages: (Canadian) English, (Argentine) Spanish, (Mandarin) Chinese, and (Jordanian/Syrian) Arabic. We employed angry, happy, sad, fearful, and neutral vocal expressions. All stimuli were part of a well-validated database of English, Spanish, Chinese, and Arabic vocal emotional stimuli^7,9,63^. A set of standardised procedures was carried out in our previous studies in adults to elicit and perceptually validate the above utterances, which express vocal emotions for each language^7,9^. As our goal in this study was to employ stimuli that would be recognized by most participants as communicating a particular emotion, we selected those vocal expressions which had the highest percentage recognition rates in our previous validation studies in adults (see Supplement 1). This approach is consistent with standardisation procedures of vocal emotion stimuli in children^28,40^. All stimuli consisted of pseudo-utterances (e.g., for English: ‘I nestered the flugs’) which mimic the phonological and morphosyntactic properties of the target language so that the emotion can only be perceived and recognised by the prosody in the speech. We deliberately selected pseudo-utterances to exclude effects of meaningful lexical-semantic information on the perception of vocally expressed emotions.

#### Vocal stimuli selection

Table 1 (see Supplement 1) presents the item by item percent recognition accuracy for the stimuli selected for this study based on previous validation studies in adults^7,9,63^. We adopted a minimal criterion of 52% correct emotion recognition based on previous studies. We selected stimuli for which recognition accuracy per emotion was significantly greater than chance (20% given five response options). Specifically, the mean recognition of the selected stimuli per language was as follows: English: 97.46%, Chinese: 93.66%, Spanish: 81.12%, Arabic: 71.90% (see^7,9^ for details). The duration of the vocal stimuli ranged between 1 and 3 seconds across languages and had a mean intensity of 70 dB. The sentences ranged between 8–14 syllables when spoken naturally to express the different emotions (for details on the stimuli acoustic properties in adult studies see^9,63^). Acoustic parameters of all the utterances used in the current study are provided in Supplementary material 4. These include the mean fundamental frequency (f0), the f0 range (maximum f0 − minimum f0) and the speech rate derived by dividing the number of syllables of each utterance by the corresponding utterance duration, in syllables per second.

#### Experimental task and procedure

The experimental paradigm consisted of a total of 4 languages (English, Spanish, Chinese, Arabic) × 5 emotion conditions (angry, happy, sad, fearful, neutral) × 2 actors (Male, Female) × 5 sentences (each sentence consisted of a different pseudo-sentence) amounting to a total of 200 trials administered in random order in two blocks of 100 trials each. There was a 5-minute break in between the two blocks. The experiment was preceded by a block of 8 practice trials, which did not appear in the experimental task, to familiarize participants with the nature of the sentences in the task. Each trial began with the presentation of a central fixation cross (500 ms), which was replaced by a blank screen and the simultaneous presentation of the vocal stimulus. The screen remained blank until the participants responded, and there was a 1000 ms interval before the onset of the next trial. The same emotional expression did not occur consecutively. Children were tested individually in a quiet room of the school. Adult participants were tested in a quiet room of the University.

Consistent with previous research in children^28,121^, the task was introduced to the children as a game. Children were told, “Children can tell how adults feel by listening to their voice. We are going to play a game about feelings. Feelings are like when you feel angry or happy. Do you know what these words mean? Do you ever feel happy? What makes you happy?” This was repeated for all emotions used in the study. This ensured that children understood the meaning of all emotion labels before taking part in the study. Following this introduction to emotions, children took part in the practice trials and the experimental task.

Participants were instructed to listen carefully to each sentence and indicate how the speaker felt based on their tone of voice by pressing a keyboard button on the computer with the verbal label ‘angry’, ‘happy’, ‘sad’, ‘scared’, and ‘neutral’. Accuracy was recorded by the computer following each trial using Psychopy software^122^. Participants were informed at the beginning of the task that the sentences were not supposed to make sense and might sound ‘foreign’ and that they should make their decision by listening carefully to the characteristics of the speaker’s tone of voice. Participants were not given any clues about the country of origin of the speaker or what language they would hear, and they did not receive any feedback about their performance accuracy. Young children were reminded to pay attention throughout the task and were given a sticker at the end of each block.

Children were given a certificate at the end of the experiment as a small ‘thank-you’ gift. Following this task, participants were asked to complete a set of questionnaires.

#### Questionnaire self-report measures

*Behavioural and emotional problems*: Children completed the hyperactivity, conduct problems, and emotional problems subscales of the Strengths and Difficulties Questionnaire (SDQ) screening questionnaire for 3-16-year-olds (Cronbach’s alpha = 0.85^123^). The SDQ is a validated self-report measure for use by 6-10-year-old children in the UK^124^. Each item is scored on a scale from 0 (not true) to 2 (certainly true). The five items for each sub-scale generate a score of 0-10. Inattention (3 items) and hyperactivity (2 items) were scored separately for the first sub-scale. Adults completed the Current Behaviour Scale measuring inattention, hyperactivity/Impulsivity (i.e., ‘I am easily distracted’; see^125^). Nine items measure inattention and 9 items measure Hyperactivity and Impulsivity. Items were scored on a 0–3 scale and scores range from 0–27 for each scale. Adults also completed the General Health Questionnaire (GHQ) measuring emotional symptoms (i.e., ‘I feel constantly under strain’; see^126^). The GHQ consists of twelve items scored either 0 or 1 and scores range from 0–12.

*Emotion regulation*: Children completed the Emotion Regulation Questionnaire for Children and Adolescents (ERQ-CA^127^). The ERQ-CA comprises of 10 items assessing the emotion regulation strategies of cognitive reappraisal (6 items) and expression suppression (4 items). Items are rated on a 5-point scale, with higher scores reflecting higher emotion regulation. The ERQ has been reported to have high internal consistency for children and adolescents (Cronbach’s alpha = 0.82 for Reappraisal, 0.75 for Suppression; see^127^ for details). The range of scores for each scale is 6–30 for the cognitive reappraisal scale (i.e., ‘when I want to feel happier about something, I change the way I am thinking about it’) and 4–20 for the expressive suppression scale (i.e., ‘when I am feeling happy I am careful not to show it’). Adults completed the corresponding Emotion Regulation Questionnaire for adults^128^. The ERQ comprises 10 items assessing cognitive reappraisal (6 items) and expressive suppression (4 items). Items are rated on a 7-point scale, with higher scores reflecting higher emotion regulation. The range of scores for each scale is 6–42 for the cognitive reappraisal scale and 4–36 for the expressive suppression scale. The ERQ has been reported to have high internal consistency (Cronbach’s alpha = 0.79 for Reappraisal, 0.73 for Suppression^128^).

*Verbal knowledge*: To ensure that the level of English was that of a native speaker, participants’ verbal knowledge was assessed with the vocabulary subtest of the Wechsler Intelligence Scale for Children (WISC-IV^129^) and the Wechsler Adult Intelligence Scale (WAIS-IV^130^) for children and adults, respectively. Words of increasing difficulty were presented orally to the participants who were required to define the words. Scores range from 0–2 based on the sophistication of the definition. Vocabulary raw scores were used in analysis. Raw scores can range between 0 and 57 for the total of 30 items for the WAIS, and between 0–68 for the total of 36 items for the WISC-IV. After converting raw scores to scaled scores (see^129,130^ for details), all participants fell within the average range of performance (see Table 6).

## Electronic supplementary material

Supplementary Information
