# Supplementary material for: The development of cross-cultural recognition of vocal emotion during childhood and adolescence
Source: Sci Rep. 2018 Jun 14;8:8659. doi: 10.1038/s41598-018-26889-1 (PMC6002529; doi:10.1038/s41598-018-26889-1)
Supplement: Supplementary file 1 — Supplementary Information [file 41598_2018_26889_MOESM1_ESM.doc]

The development of cross-cultural recognition of vocal emotion during childhood and adolescence

Georgia Chronaki, PhD^1, 2,3^, Michael Wigelsworth, PhD^4^, Marc D. Pell, PhD^5^ & Sonja A. Kotz, PhD^2,6,7^

1. Developmental Cognitive Neuroscience (DCN) Laboratory, School of Psychology, University of Central Lancashire, UK.
2. Division of Neuroscience & Experimental Psychology, University of Manchester, UK.
3. School of Psychology, University of Southampton, Southampton, UK.
4. Manchester Institute of Education, University of Manchester, UK.
5. School of Communication Sciences and Disorders, McGill University, Montréal, QC, Canada.
6. Department of Neuropsychology, Max-Planck Institute for Human Cognitive and Brain Sciences Leipzig, Germany.
7. Faculty of Psychology and Neuroscience, Department of Neuropsychology and Psychopharmacology, Maastricht University, Netherlands.

Corresponding author:

Georgia Chronaki, PhD

Developmental Cognitive Neuroscience (DCN) Laboratory

School of Psychology

University of Central Lancashire

Preston PR1 2HE

United Kingdom

Email: GChronaki@uclan.ac.uk

**Supplement 1**

*Table 1. Item by item % agreement on the vocal stimuli selected for this study based on previous validation studies in adults (data from Pell et al., 2009; Pell, Monetta, Paulmann & Kotz, 2009; Liu & Pell, 2012).*

|  | English | | Chinese | | Spanish | | Arabic | |
| --- | --- | --- | --- | --- | --- | --- | --- | --- |
|  | Male | Female | Male | Female | Male | Female | Male | Female |
| **Angry** |  |  |  |  |  |  |  |  |
| Item 1 | 100 | 100 | 100 | 74 | 95 | 100 | 59 | 82 |
| Item 2 | 100 | 100 | 100 | 87 | 90 | 95 | 59 | 82 |
| Item 3 | 100 | 100 | 100 | 91 | 90 | 90 | 59 | 82 |
| Item 4 | 100 | 100 | 100 | 91 | 90 | 90 | 76 | 82 |
| Item 5 | 100 | 100 | 100 | 91 | 90 | 90 | 76 | 88 |
| **Sad** |  |  |  |  |  |  |  |  |
| Item 1 | 100 | 91 | 91 | 100 | 76 | 100 | 76 | 76 |
| Item 2 | 100 | 96 | 96 | 91 | 57 | 95 | 76 | 71 |
| Item 3 | 100 | 96 | 96 | 91 | 52 | 81 | 82 | 71 |
| Item 4 | 100 | 96 | 96 | 91 | 48 | 81 | 65 | 76 |
| Item 5 | 100 | 100 | 100 | 96 | 43 | 76 | 65 | 94 |
| **Happy** |  |  |  |  |  |  |  |  |
| Item 1 | 83 | 91 | 91 | 87 | 100 | 95 | 41 | 47 |
| Item 2 | 92 | 96 | 96 | 87 | 100 | 90 | 59 | 47 |
| Item 3 | 92 | 96 | 96 | 87 | 100 | 90 | 53 | 76 |
| Item 4 | 96 | 87 | 87 | 91 | 100 | 86 | 65 | 71 |
| Item 5 | 83 | 87 | 87 | 100 | 100 | 81 | 82 | 53 |
| **Fear** |  |  |  |  |  |  |  |  |
| Item 1 | 100 | 91 | 91 | 83 | 95 | 86 | 82 | 82 |
| Item 2 | 100 | 96 | 96 | 91 | 95 | 86 | 76 | 59 |
| Item 3 | 96 | 96 | 96 | 96 | 90 | 81 | 71 | 65 |
| Item 4 | 100 | 96 | 96 | 96 | 90 | 81 | 65 | 71 |
| Item 5 | 100 | 100 | 100 | 87 | 86 | 81 | 65 | 76 |
| **Neutral** |  |  |  |  |  |  |  |  |
| Item 1 | 92 | 100 | 100 | 91 | 100 | 38 | 76 | 71 |
| Item 2 | 92 | 100 | 100 | 91 | 95 | 24 | 94 | 76 |
| Item 3 | 92 | 100 | 100 | 91 | 95 | 24 | 82 | 71 |
| Item 4 | 96 | 100 | 100 | 91 | 95 | 24 | 82 | 82 |
| Item 5 | 91 | 100 | 100 | 96 | 95 | 24 | 76 | 82 |

**Supplement 2**

The language effect varied by emotion type (*F* (9, 684) ^language x emotion^  = 88.70, *p* < .001, *η^2^_p_* =.54). Vocal expressions of anger compared to fear were significantly more accurately recognised in English than in Arabic. Expressions of fear, which were recognized relatively poorly when compared to other emotions in other languages, were significantly more accurately recognised in Arabic. In addition, fear compared to happiness was significantly more accurately recognised in Chinese than English. Vocal expressions of happiness compared to fear were significantly more accurately recognised in Spanish than in Chinese (all ps<001).

**Supplement 3**

We run Pearson’s correlations examining associations between recognition accuracy for each emotional expression for each language and behavioural and emotional symptoms in the whole sample of children and adults separately. These analyses controlled for age because age was significantly associated with recognition accuracy for native and non-native language (r=.56, p<.001). Results showed that conduct problems were negatively associated with accuracy for English angry (r=-.33, p=.010) and happy (r=-.35, p=.008) voices. In addition, accuracy for angry Chinese voices was negatively associated with child hyperactivity (r=-.30, p=.024) and emotional problems (r=-.32, p=.016). Accuracy for sad Arabic voices was negatively correlated with child hyperactivity (r=-.32, p=.017) and conduct problems (r=-.40, p=.002). Emotional problems in children were negatively associated with accuracy for happy Spanish voices (r=-.34, p=.010) and sad Chinese voices (r=-.30, p=.020). Emotional problems in children were also negatively associated with accuracy for neutral Chinese voices (r=-.36, p=.006). In adults, emotional problems were negatively associated with accuracy for sad Chinese voices (r=-.46, p=.030) and fearful Chinese voices (r=-.60, p=.008). Cognitive appraisal in children was positively associated with accuracy for English angry voices (r=.30, p=.015), angry Chinese voices (r=.30, p=.016) and neutral Chinese voices (r=.33, p=.015). Accuracy for happy Arabic voices was negatively associated with cognitive reappraisal in children (r=-.30, p=.020) and adults (r=-.48, p=.040). Expressive suppression in children was negatively associated with accuracy for fearful Arabic voices (r=-.30, p=.015).

**Supplement 4**

Table 2a. Acoustic parameters (mean f0, range f0 and speech rate) of the vocal stimuli item by item used in the study for English and Spanish.

|  | English | | | | | | Spanish | | | | | | |
| --- | --- | --- | --- | --- | --- | --- | --- | --- | --- | --- | --- | --- | --- |
|  | Male | | | Female | | | Male | | | | Female | | |
|  | meanf0 | rangef0 | speech rate | meanf0 | range f0 | speech rate | | meaf0 | range f0 | speech rate | meanf0 | rangef0 | speech rate |
| **Angry** |  |  |  |  |  |  | |  |  |  |  |  |  |
| Item 1 | 254 | 199.39 | 3.64 | 224 | 128.70 | 3.26 | | 165 | 173.90 | 4.39 | 159 | 374.13 | 3.66 |
| Item 2 | 230 | 195.99 | 4.16 | 240 | 206.00 | 3.39 | | 176 | 404.37 | 5.00 | 158 | 361.26 | 5.62 |
| Item 3 | 204 | 210.56 | 2.68 | 188 | 77.40 | 3.40 | | 169 | 183.98 | 5.48 | 166 | 289.80 | 4.40 |
| Item 4 | 220 | 225.35 | 3.75 | 189 | 104.57 | 4.18 | | 167 | 191.26 | 4.58 | 185 | 104.86 | 5.80 |
| Item 5 | 223 | 201.90 | 4.48 | 210 | 172.20 | 3.80 | | 189 | 413.90 | 4.94 | 163 | 139.75 | 5.12 |
| **Sad** |  |  |  |  |  |  | |  |  |  |  |  |  |
| Item 1 | 130 | 62.75 | 4.28 | 210 | 67.90 | 3.56 | | 105 | 392.70 | 5.65 | 155 | 401.85 | 3.80 |
| Item 2 | 110 | 57.49 | 4.25 | 206 | 64.28 | 4.10 | | 113 | 70.20 | 5.18 | 158 | 416.54 | 4.70 |
| Item 3 | 148 | 93.49 | 4.06 | 218 | 50.20 | 4.49 | | 119 | 359.67 | 6.20 | 165 | 400.40 | 4.65 |
| Item 4 | 137 | 134.97 | 4.52 | 240 | 87.80 | 3.10 | | 110 | 401.10 | 4.83 | 165 | 384.46 | 5.26 |
| Item 5 | 144 | 79.67 | 4.46 | 238 | 94.90 | 3.48 | | 100 | 56.50 | 3.43 | 152 | 98.14 | 5.55 |
| **Happy** |  |  |  |  |  |  | |  |  |  |  |  |  |
| Item 1 | 149 | 64.30 | 4.39 | 187 | 107.60 | 5.55 | | 193 | 212.40 | 4.65 | 265 | 315.99 | 5.20 |
| Item 2 | 165 | 98.34 | 3.93 | 182 | 83.70 | 4.80 | | 176 | 396.38 | 4.25 | 226 | 335.67 | 5.30 |
| Item 3 | 138 | 64.66 | 5.12 | 227 | 174.97 | 3.84 | | 193 | 221.30 | 4.80 | 220 | 258.36 | 5.37 |
| Item 4 | 155 | 69.46 | 5.04 | 180 | 65.87 | 5.29 | | 184 | 169.80 | 4.27 | 217 | 223.64 | 5.52 |
| Item 5 | 149 | 79.36 | 3.46 | 193 | 131.68 | 4.10 | | 190 | 186.65 | 5.20 | 232 | 184.95 | 5.44 |
| **Fear** |  |  |  |  |  |  | |  |  |  |  |  |  |
| Item 1 | 237 | 130.15 | 6.46 | 350 | 147.67 | 4.10 | | 92 | 51.35 | 6.62 | 215 | 170.10 | 6.06 |
| Item 2 | 260 | 133.08 | 6.05 | 329 | 219.90 | 5.50 | | 110 | 57.30 | 6.06 | 219 | 328.94 | 5.55 |
| Item 3 | 275 | 126.20 | 6.01 | 343 | 136.29 | 7.00 | | 126 | 310.30 | 6.00 | 244 | 290.29 | 4.80 |
| Item 4 | 264 | 125.82 | 5.89 | 363 | 154.70 | 5.88 | | 137 | 76.40 | 5.95 | 240 | 294.04 | 4.67 |
| Item 5 | 260 | 167.73 | 5.74 | 310 | 113.20 | 6.45 | | 144 | 403.90 | 5.10 | 215 | 311.00 | 6.55 |
| **Neutral** |  |  |  |  |  |  | |  |  |  |  |  |  |
| Item 1 | 136 | 36.50 | 4.89 | 188 | 108.00 | 4.56 | | 94 | 42.23 | 5.48 | 136 | 92.12 | 5.55 |
| Item 2 | 120 | 49.28 | 5.19 | 194 | 163.70 | 5.25 | | 106 | 325.20 | 4.25 | 120 | 40.83 | 4.78 |
| Item 3 | 129 | 54.88 | 5.53 | 206 | 96.40 | 5.17 | | 107 | 56.40 | 3.90 | 129 | 80.60 | 3.90 |
| Item 4 | 129 | 40.72 | 6.16 | 189 | 117.47 | 4.30 | | 105 | 160.78 | 4.00 | 135 | 398.42 | 4.42 |
| Item 5 | 125 | 44.70 | 5.59 | 190 | 84.67 | 4.95 | | 104 | 51.94 | 5.10 | 122 | 392.50 | 4.74 |

Table 2b. Acoustic parameters (mean f0, range f0 and speech rate) of the vocal stimuli item by item used in the study for Chinese and Arabic.

|  | Chinese | | | | | | Arabic | | | | | | |
| --- | --- | --- | --- | --- | --- | --- | --- | --- | --- | --- | --- | --- | --- |
|  | Male | | | Female | | | Male | | | | Female | | |
|  | meanf0 | rangef0 | speech rate | meanf0 | range f0 | speech rate | | meaf0 | range f0 | speech rate | meanf0 | rangef0 | speech rate |
| **Angry** |  |  |  |  |  |  | |  |  |  |  |  |  |
| Item 1 | 239 | 214.16 | 7.15 | 346 | 259.90 | 4.95 | | 150 | 105 | 5.40 | 197 | 95 | 4.40 |
| Item 2 | 278 | 145.40 | 7.53 | 396 | 247.64 | 6.30 | | 138 | 90 | 6.23 | 188 | 72 | 3.67 |
| Item 3 | 251 | 303.45 | 7.45 | 281 | 293.56 | 6.39 | | 165 | 95 | 4.93 | 204 | 105 | 3.92 |
| Item 4 | 261 | 221.20 | 6.64 | 289 | 276.53 | 5.85 | | 153 | 110 | 6.14 | 217 | 192 | 4.98 |
| Item 5 | 274 | 126.28 | 8.25 | 369 | 224.17 | 7.60 | | 155 | 145 | 6.40 | 206 | 66 | 4.80 |
| **Sad** |  |  |  |  |  |  | |  |  |  |  |  |  |
| Item 1 | 179 | 82.16 | 6.10 | 246 | 76.90 | 4.09 | | 136 | 38 | 4.78 | 208 | 73 | 5.00 |
| Item 2 | 182 | 225.20 | 5.45 | 232 | 118.37 | 4.46 | | 117 | 20 | 5.00 | 252 | 70 | 4.45 |
| Item 3 | 188 | 77.80 | 6.12 | 235 | 324.78 | 5.13 | | 120 | 17 | 5.47 | 219 | 115 | 4.39 |
| Item 4 | 191 | 57.95 | 5.35 | 220 | 296.29 | 4.50 | | 112 | 35 | 4.77 | 216 | 63 | 5.10 |
| Item 5 | 189 | 85.00 | 4.90 | 234 | 108.87 | 4.48 | | 101 | 30 | 4.94 | 219 | 48 | 5.36 |
| **Happy** |  |  |  |  |  |  | |  |  |  |  |  |  |
| Item 1 | 254 | 192.63 | 6.20 | 330 | 315.20 | 5.30 | | 163 | 209 | 5.10 | 219 | 182 | 4.07 |
| Item 2 | 254 | 241.28 | 5.72 | 343 | 241.80 | 5.03 | | 209 | 168 | 5.83 | 244 | 200 | 4.08 |
| Item 3 | 270 | 205.20 | 6.25 | 330 | 230.26 | 6.49 | | 176 | 196 | 5.09 | 267 | 201 | 4.18 |
| Item 4 | 249 | 310.76 | 7.12 | 312 | 337.63 | 5.30 | | 208 | 120 | 4.58 | 264 | 214 | 4.19 |
| Item 5 | 245 | 281.76 | 6.73 | 336 | 387.40 | 6.55 | | 218 | 150 | 4.75 | 224 | 142 | 4.40 |
| **Fear** |  |  |  |  |  |  | |  |  |  |  |  |  |
| Item 1 | 236 | 136.60 | 6.28 | 268 | 141.60 | 6.49 | | 146 | 17 | 6.00 | 350 | 118 | 5.30 |
| Item 2 | 222 | 305.79 | 6.33 | 262 | 150.75 | 6.00 | | 193 | 80 | 5.99 | 391 | 160 | 4.50 |
| Item 3 | 249 | 127.19 | 6.48 | 263 | 141.85 | 5.87 | | 209 | 109 | 7.77 | 390 | 210 | 5.54 |
| Item 4 | 198 | 145.60 | 6.20 | 276 | 167.00 | 5.28 | | 236 | 105 | 6.95 | 249 | 99 | 7.05 |
| Item 5 | 225 | 77.39 | 6.09 | 220 | 340.50 | 5.60 | | 204 | 35 | 8.30 | 305 | 124 | 5.70 |
| **Neutral** |  |  |  |  |  |  | |  |  |  |  |  |  |
| Item 1 | 127 | 91.00 | 5.92 | 221 | 116.10 | 5.75 | | 156 | 95 | 5.78 | 203 | 72 | 4.88 |
| Item 2 | 136 | 101.99 | 6.13 | 217 | 144.77 | 5.38 | | 137 | 76 | 6.20 | 212 | 154 | 4.46 |
| Item 3 | 123 | 38.79 | 6.64 | 206 | 347.99 | 5.95 | | 158 | 108 | 5.04 | 229 | 178 | 4.98 |
| Item 4 | 116 | 69.07 | 7.38 | 216 | 296.40 | 6.20 | | 140 | 90 | 6.09 | 217 | 89 | 4.70 |
| Item 5 | 129 | 89.18 | 7.02 | 224 | 225.90 | 5.07 | | 146 | 111 | 7.47 | 214 | 105 | 4.30 |

Table 3a. Acoustic parameters (mean f0, range f0 and speech rate) of the vocal stimuli per emotion used in the study for English and Spanish.

|  | English | | | | | | Spanish | | | | | | |
| --- | --- | --- | --- | --- | --- | --- | --- | --- | --- | --- | --- | --- | --- |
|  | Male | | | Female | | | Male | | | | Female | | |
|  | meanf0 | rangef0 | speech rate | meanf0 | range f0 | speech rate | | meaf0 | range f0 | speech rate | meanf0 | rangef0 | speech rate |
| Angry | 226 | 206.64 | 3.74 | 210 | 137.77 | 3.61 | | 174 | 273.48 | 4.878 | 166 | 253.96 | 4.92 |
| Happy | 134 | 85.67 | 4.31 | 222 | 73.01 | 3.76 | | 110 | 256.04 | 5.06 | 159 | 340.27 | 4.79 |
| Sad | 151 | 75.22 | 4.38 | 194 | 112.76 | 4.722 | | 187 | 237.31 | 4.636 | 232 | 263.72 | 5.36 |
| Fear | 259 | 136.59 | 6.03 | 339 | 154.35 | 5.794 | | 122 | 179.86 | 5.95 | 227 | 278.87 | 5.52 |
| Neutral | 128 | 45.22 | 5.47 | 194 | 114.04 | 4.848 | | 103 | 127.316 | 4.55 | 129 | 200.89 | 4.67 |

Table 3b. Acoustic parameters (mean f0, range f0 and speech rate) of the vocal stimuli per emotion used in the study for Chinese and Arabic.

|  | Chinese | | | | | | Arabic | | | | | | |
| --- | --- | --- | --- | --- | --- | --- | --- | --- | --- | --- | --- | --- | --- |
|  | Male | | | Female | | | Male | | | | Female | | |
|  | meanf0 | rangef0 | speech rate | meanf0 | range f0 | speech rate | | meaf0 | range f0 | speech rate | meanf0 | rangef0 | speech rate |
| Angry | 261 | 202.10 | 7.40 | 336 | 260.36 | 6.22 | | 152 | 109.00 | 5.82 | 202 | 106.00 | 4.36 |
| Happy | 186 | 105.60 | 5.58 | 234 | 185.04 | 4.53 | | 117 | 28.40 | 4.99 | 222 | 73.80 | 4.87 |
| Sad | 255 | 246.30 | 6.40 | 330 | 302.46 | 5.73 | | 194 | 168.60 | 5.07 | 243 | 187.80 | 4.18 |
| Fear | 226 | 158.50 | 6.27 | 258 | 188.34 | 5.84 | | 197 | 69.20 | 7.01 | 337 | 142.80 | 5.62 |
| Neutral | 126 | 78.00 | 6.61 | 217 | 226.24 | 5.67 | | 147 | 96.40 | 6.11 | 215 | 119.60 | 4.67 |
